# Association of stress management skills and stressful life events with allergy risk: a case-control study in southern China

**DOI:** 10.1186/s12889-021-11333-3

**Published:** 2021-06-30

**Authors:** Jingru Cheng, Fei Li, Yigui Lai, Jieyu Chen, Xiaomin Sun, Lei Xiang, Pingping Jiang, Shengwei Wu, Ya Xiao, Lin Zhou, Ren Luo, Xiaoshan Zhao, Yanyan Liu

**Affiliations:** 1 Department of Nephrology, the First Affiliated Hospital of Zhengzhou University, Zhengzhou, 450052 China; 2 School of Traditional Chinese Medicine, Southern Medical University, Guangzhou, 510515 Guangdong China; 3 Department of Traditional Chinese Medicine, People’s Hospital of Yangjiang, Yangjiang, 529500 Guangdong China; 4 Endocrinology Department, Nanfang Hospital, Southern Medical University, Guangzhou, 510515 Guangdong China

**Keywords:** Stressful life events, Stress management skills, Allergy risk

## Abstract

**Background:**

Psychosocial stress and stressful life events are known to aggravate allergic diseases. Less is known about the impact of stress management skills on allergies. Here we sought to determine whether stress management skills are associated with the allergies and to assess the combined effects of stress management skills and stressful events on allergy risk.

**Methods:**

A survey on risk factors for self-reported allergic diseases was carried out among 28,144 southern Chinese people; 14 stressful life events and 8 stress management skills were retrospectively recorded in a case-control setting with multivariate logistic regression analysis. Multiplicative and additive interactions between stressful events and stress management skills were evaluated.

**Results:**

Stressful events significantly increased allergy risk. The odds ratio (OR) for allergies was 1.65 (95% confidence interval CI, 1.41–1.93) for those reporting one or two stressful events and 3.10 (95% CI, 2.55–3.79) for those reporting more than three stressful events compared to participants without stressful events. Stress management skills were adversely associated with allergic risk for people experiencing stressful events (OR, 0.71; 95% CI, 0.53–0.97) when adjusted demographically, particularly “concentrate on pleasant thoughts at bedtime” (OR, 0.67; 95% CI, 0.51–0.89), “pace myself to prevent tiredness” (OR, 0.67; 95% CI, 0.54–0.83), “get enough sleep” (OR, 0.48; 95% CI, 0.32–0.72) and “take some time for relaxation each day” (OR, 0.55; 95% CI, 0.37–0.80). But in people without stressful events, no association was observed. There was a significant linear trend for allergy risk from good stress management skills with no stressful events to poor stress management skills with stressful events (*P* < 0.001), with significant interaction in additive models (*P* = 0.006).

**Conclusions:**

There are independent and antagonistic combined associations of stressful life events and stress management skills with allergy risk. The data supports the use of stress management skills in managing allergic disease among people with stressful life events.

**Supplementary Information:**

The online version contains supplementary material available at 10.1186/s12889-021-11333-3.

## Background

Allergic diseases have risen dramatically over the last decades [1] and become a worldwide burden in different population. In China, recent data show a 6.5% increase in the prevalence of allergic rhinitis from 2005 to 2011 [2], and a 150% growth of the asthma prevalence in children (from approximately 2% in 1990 to more than 3% in 2020) [3]. Besides the common risk factors such as genetics [4], environment [5], female [6] and lifestyle factors [7], psychosocial stress (means the self-reported sensation of tension, irritability, nervousness, anxiety or sleeplessness [8] associated with poor health, family relationships, living arrangements, finance, work and stressful life events) is becoming more and more important in the onset or exacerbations of allergic diseases [9–12]. Possible mechanisms are regulation of epigenetics [13], or production of cytokines affecting neuroimmune processes, autonomic balance and inflammation [14–17], or activation of the hypothalamic-pituitary-adrenal (HPA) axis with anti-inflammatory and bronchodilator effects [18]. This relationship linking psychosocial stress, central nervous system, and alterations in immune and endocrine function has been known as psychoneuroimmunology [19].

Stress has been described as “a constellation of events, comprised of a stimulus (stressor) that precipitates a reaction in the brain” [20]. Critical events, such as disease or the death of a parent or friend, conflicts in personal relationships, or changes in living environment, are stressors in life [21]. They demand psychological readjustment and may have a greater impact on the susceptibility or the course of the diseases [22]. The association between stressful events and asthma attacks was suggested in an early research [23], the author divided one hundred adult asthmatic patients into 4 groups by age at onset(0–16,17-27,28–35 and 36–48 years), underwent a psychiatric interview and psychological investigations, the results indicate the onset age of asthma may be the end-product of somatic, psychic and psychosocial factors. Although the study did not focus on stressful life events, it did include some events associated with asthma development such as separation from a close relative or friend/occupational stress, economic difficulties, environment in adulthood et al. In addition, another study [24] showed an immediate effect in the first 2 days after experience a severe negative life event, with a 4.69-fold increase in the risk of a new asthma attack(p<0.001). Several other retrospective studies [25–28] and at least two prospective study [29, 30] have reported a positive relationship between stressful events and the incidence of allergic disease among adolescents and adults. Besides above mentioned psychoneuroimmunology mechanism, G Herberth et al. [31] provide excellent evidence that neuropeptide vasoactive intestinal peptide might act as mediators between stressful life events and immune regulation in children with separated/divorced parents.

It follows that managing stress could be expected to have salutary effects on the occurrence of allergic diseases and underlying disease course. Stress-reducing interventions aim at modifying stress appraisal and decreasing subjective anxiety. Stress management skills are lifestyle behaviors that help people to prevent stressful events from occurring and manage their responses to those stressful events. There are indications that stress management interventions might affect basal autonomic or endocrine responses [32], and immune responses [33]. Walker et al. [34] found that patients who received relaxation training and image-guided therapy prior to chemotherapy reported a better quality of life and less emotional distress than patients who did not. In a systematic review, Huntley et al. [35] found that relaxation therapy had a positive effect on asthma outcomes. However, the specific relationship between stress management skills and the occurrence of allergic diseases, and its combined effect with stressful events on allergies, has largely been unexplored.

This case-control study sought to examine the link between stress management skills and allergies in a southern Chinese population, as well as it’s interactional effects with stressful events on allergic diseases. It was hypothesized that better stress management skills would be associated with lower allergy risk and would moderate the negative influence of stressful events on allergy risk.

## Methods

### Study design and subjects

A survey on possible risk factors for the development of asthma and allergies was undertaken between April 2012 and January 2013 in six cities randomly selected in Guangdong province, southern China (Guangzhou, Huizhou, Shaoguan, Jiangmen, Zhanjiang and Heyuan). We selected one or two convenient areas (e.g. schools, companies, government agencies or factories) in each city for cluster sampling. Original questionnaire data were obtained from 28,144 persons. Of these, 13,491 (47.94%) were men and 14,653 (52.06%) women. A matched case-control study was conducted to investigate the relationship between life events, stress management skills and allergic diseases. Case and control participants were selected from these 28,144 respondents. The case participants were willing to participate and met the following inclusion criteria: (i) 18 years of age or older; (ii) with one or more self-reported allergic diseases (asthma or bronchitis, allergic rhinitis, atopic dermatitis); (iii) not pregnant or lactating, and (iv) no critical illness (eg. depression/anxiety, DM, hypertension, cardiovascular or renal diseases, et al.) or intake of medication in the previous 2 weeks.

Self-reported allergic diseases were selected by positive answers to any of the following questions (Questionnaire 1):

- Asthma: Have you ever had asthma? If so, has a doctor diagnosed the disease?
- Allergic rhinitis: Have you ever had ‘hay fever’ or other allergic nasal symptoms (sneezing, nasal itching, blocked nose or runny nose) in the absence of a cold or flu, e.g. from pollen or animals? A doctor’s diagnosis for the disorder was required.
- Atopic dermatitis: Have you ever had symptoms of itchy rash called atopic eczema or eczema localized to flexural regions (such as folds of the elbows, behind the knees), facial, or generalized to the body? In addition, a doctor’s diagnosis was asked about.

For each case participant, we validate the allergic diseases by asking “has a doctor diagnosed the disease” or by evaluating their medical histories getting from each unit managers (at least 1 year medical examination report between 2012 and 2013 for each participant). Cases who responsed above questions “yes” or had “allergic disease” medical record met the criteria for self-reported allergic diseases, and were included in the case-control study. Two matched control participants were randomly selected from the respondents who had reported no symptoms suggestive of allergic diseases during their lifetime, and were incidence-density matched to the case participants by departments of the selected area, sex and age (±3 years). Eligible control participants were 18 years or older, not pregnant or lactating, and had no history of any other serious chronic disease. As is standard in incidence-density matching, a control participant could serve as a control for more than one case participant [36]. A total of 1340 participants with allergies and 2662 healthy control participants were recruited.

### Questions on stressful life events

We asked if the respondents had ever experienced various specific potentially stressful life events (Questionnaire 1). The events were based on a commonly used life event scale [37]. The original list included 14 items, and subjects were asked to respond either “yes (scored 1)” or “no (scored 0)”. Based on preliminary data analysis, and knowledge of life events generally regarded as stressful, the events were divided into three groups: disease or death of family members or close friends; conflicts in personal relationships (including relationships with family members, spouses, colleagues or friends); life changes or other events (e.g. economic, career, lifestyle, living environment). Indicators used in the analyses included a total stressful life event score (range from 0 to 14) obtained by summing all 14 items as well as subscale scores related to particular domains (Table 2).

### Assessment of stress management skills

The Health-Promoting Lifestyle Profile II (HPLP-II) was used to measure stress management skills (Questionnaire 2). The scale was developed by Walker and colleagues in 1987 [38] and later revised as the HPLP-II [39]. The Chinese version of HPLP-II was developed by Lee and Loke, who established validity and credibility with an internal consistency coefficient (Cronbach’s alpha) of 0.91 [39, 40]. It measures six dimensions of self-reported health-promoting behaviors, including spiritual growth (nine items), health responsibility (nine items), physical activity (eight items), nutrition (nine items), interpersonal relations (nine items) and stress management (eight items). Each subgroup can be used independently [41]. In this study, we only used the stress management subscale, the Cronbach’s alpha of which was reported in Hong Kong as 0.75 [42]. It includes eight items (Table S1), covers most approaches of managing or reducing stress. Participants were asked to rate the frequency of stress management behaviors using the four-point Likert scale as 1 (never or rarely), 2 (sometimes), 3 (often), 4 (routinely). The total stress management skill score were all 8 items score combined, ranged from 8 to 32, higher scores represent more engagement in stress management behaviour. For descriptive scores and logistic regression analysis, the total stress management skill ratings were trichotomized as good (25–32), moderate (17–24) or poor (8–16).

### Data collection and other exposure

To increase participation and ensure the completeness and truthfulness of each questionnaire, recruitment was conducted in conference halls of different selected units by trained investigators in cooperation with the administrators. The self-administered questionnaire included an introduction detailing the objectives of the study and guaranteeing anonymity and confidentiality of the data. Further questions on demographic factors (age, sex, height, body weight and education), active and passive smoking history, alcohol drinking habits, physical activity, history of allergic disease, stressful life events and stress management skills were surveyed.

Educational level was categorized into three groups: (i) junior high school education (compulsory schooling), (ii) high school education, and (iii) any university, college or higher education by reported highest academic background at baseline. Body mass index was calculated as weight in kilograms divided by the square of height in meters. We categorized body mass index as < 18.5 (malnourished), 18.5–23.9 (normal weight), or ≥ 24 (overweight), which differs from the World Health Organization classification [43] but is suitable for Chinese adult populations [44]. Information on smoking was based on questions regarding never smoking, currently smoking and ever having smoked. Participants were asked to state how often they drank alcohol and engaged in physical activity (never, sometimes, often, always) through the questions, “Do you normally drink alcohol more than three times per week?” and “Do you exercise vigorously for 20 min or more at least three times a week (such as brisk walking, bicycling, aerobic dancing, using a stair climber)?”

### Statistical analysis

For demographic and exposure variables, differences in means or proportions between participants with and without allergic diseases were evaluated using Student’s *t* or chi-squared tests, as appropriate. The risks of allergies with exposure to stressful events and stress management skills were analyzed using conditional logistic regression models. The proportion of stressful events was used to predict allergies outcomes; in addition, the total number of life events was categorized as 0, 1–2 and ≥ 3. For stress management skills, stratified analyses were conducted among subgroups with or without stressful events. The logistic regression model included the following potential confounders: age, sex, education, body mass index, smoking status, alcohol intake and physical activity. The interaction effect between stressful events and stress-management skills was further evaluated by multiplicative and additive models. We tested for multiplicative interaction by including the product term in multivariate logistic regression. Additive interaction was assessed using the method of Rothman [45].

The 95% confidence intervals (95% CI) for the odds ratios (ORs) were calculated; *P* < 0.05 was considered to be statistically significant. All statistical analyses were performed using SPSS Statistics for Windows, version 13.0 (SPSS Inc., Chicago, IL, USA) and SAS software 9.3 (SAS Inc., Cary, N.C., USA).

## Results

Demographic characteristics, smoking status, alcohol intake and physical activity are showed in Table 1. Among participants without self-reported illness or injury group, cases were likely to take less vigorous exercise (*P* < 0.001). Alcohol intake, educational level, body mass index and smoking status did not have significant association with allergic diseases in participants either with or without self-reported illness or injury (*P* > 0.05).

**Table 1:** **Basic characteristics of 1340 cases and 2662 controls in southern China**

Variable	participants without self-reported illness or injury^a^		P	participants with self-reported illness or injury^a^		P
	Cases N(%)	Controls N(%)		Cases N(%)	Controls N(%)	
**Age (year, mean ± SD)**	28.25 ± 9.46	28.10 ± 9.26	0.658	26.90 ± 8.14	25.63 ± 7.80	0.104
**Sex**			0.528			0.165
Male	509(46.0)	1110(44.9)		92(39.3)	87(46.0)	
Female	597(54.0)	1363(55.1)		142(60.7)	102(54.0)	
**Education**			0.829			0.953
Compulsory school	73(6.6)	177(7.2)		10(4.3)	7(41.2)	
High school graduate	341(30.8)	754(30.5)		57(24.4)	47(24.9)	
University/college degree	692(62.6)	1542(62.4)		167(71.4)	135(71.4)	
**BMI (kg/m**^**2**^**)**			0.062			0.895
<18.5	187(16.9)	486(19.7)		52(22.2)	40(21.2)	
18.5–23.9	734(66.4)	1628(65.8)		151(64.5)	126(66.7)	
≥ 24	185(16.7)	359(14.5)		31(13.2)	23(12.2)	
**Smoking status**			0.113			0.626
Never	945(85.4)	2161(87.4)		207(88.5)	170(89.9)	
Current/Former	161(14.6)	312(12.6)		27(11.5)	19(10.1)	
**Alcohol intake**			0.145			0.096
Never	324(29.3)	804(32.5)		53(22.6)	53(28.0)	
Sometimes	757(68.4)	1621(65.5)		174(74.4)	135(43.7)	
Often/always	25(2.3)	48(1.9)		7(3.0)	1(0.5)	
**Physical activity**^**b**^			**<0.001**			0.185
Rarely/never	275(24.9)	454(18.4)		58(24.8)	33(17.5)	
Sometimes	483(43.7)	1049(42.4)		96(41.0)	87(46.0)	
Often/always	348(31.5)	970(39.2)		80(34.2)	69(36.5)	

^a^Self-reported illness or injury: personal injury or illness as one kind of life event, but not serious because people with serious or chronic diseases are excluded at the beginning of the research. ^b^Vigorous physical activity in leisure-time exercise. Values of *P* for age were calculated using the independent-samples t test; and others using the chi-squared test. Significant differences are highlighted in bold

### Stressful life events

The percentage of subjects experienced stressful events is shown in Table 2. Overall, 65.8% of participants reported one or more stressful events during their lifetime. Life changes or other events, e.g., overload, economic plight, career change, or living environment change, were reported by 62.2% of adults, which were more prevalent than disease or death or conflicts that were reported by 22.3 and 29.3% of participants, respectively. People with allergies were significantly more likely to report a wide variety of stressful life events (*P* < 0.05), except for “death of a family member or close friend” and “career change”. And for the three bigger classification of the events, which means “disease or death, conflicts in personal relationships and life changes or other events”, the differences between case and control participants are also statistically significant (*P* < 0.001).

**Table 2:** Percents of stressful life events in case-control participants and in all participants

Self-reported events N (%)	Case participants (*n* = 1340)	Control participants (*n* = 2662)	P*	All participants (*n* = 25,938)^a^
**Disease or death**	328 (24.5)	346 (13.0)	**<0.001**	5786 (22.3)
Personal injury or illness	234 (17.5)	189 (7.1)	**<0.001**	2590 (10.0)
Severe disease of family member or close friend	82 (6.1)	83 (3.1)	**<0.001**	1133 (4.4)
Death of family member or close friend	68 (5.1)	106 (4.0)	0.110	953 (3.7)
**Conflicts in personal relationships**	396 (29.6)	533 (20.0)	**<0.001**	7587 (29.3)
Interpersonal disharmony	150 (11.2)	176 (6.6)	**<0.001**	2039 (7.9)
Family discord	59 (4.4)	72 (2.7)	**0.004**	771 (3.0)
Marital disruption	30 (2.2)	32 (1.2)	**0.012**	378 (1.5)
Trouble from children	91 (6.8)	116 (4.4)	**0.001**	1400 (5.4)
Split up from boyfriend or girlfriend	212 (15.8)	290 (10.9)	**<0.001**	3040 (11.7)
**Life changes or other events**	832 (62.1)	1343 (50.0)	**<0.001**	16,136 (62.2)
Overwork	561 (41.9)	814 (30.6)	**<0.001**	8672 (33.4)
Economic plight	288 (21.5)	464 (17.4)	**0.002**	5554 (21.4)
Career change	46 (3.4)	63 (2.4)	0.051	1030 (4.0)
Living environment change	221 (16.5)	365 (13.7)	**0.019**	3990 (15.4)
Lifestyle change	191 (14.3)	306 (11.5)	**0.013**	3444 (13.3)
Suffer a criminal or civil penalty	3 (0.2)	0 (0.0)	**0.015**	17 (0.1)
**Total stressful events**				
Any stressful event	1000(74.6)	1595(59.9)	**<0.001**	17,075(65.8)
Number of stressful events			**<0.001**	
0	340 (25.4)	1067 (40.1)		8863 (34.2)
1 or 2	663 (49.5)	1251 (47.0)		12,498 (48.2)
≥ 3	337 (25.1)	344 (12.9)		4577 (17.6)

* Values of *P* for comparison of people with and without allergies were calculated using the chi-squared test. Significant differences are highlighted in bold. ^a^Subjects over 18 years old

Relationship between stressful events and allergies was explored in 4002 southern Chinese people (1340 case participants and 2662 control participants). Allergies were significantly associated with the number of total stress events, disease or death events, conflicts and life changes or other events. These associations were only slightly attenuated after adjustment for age, sex, education, body mass index, smoking status, alcohol intake and physical activity (Table 3). A dose-response relationship was found between allergies and increasing total stressful events, the data showed that the risk of allergies was significantly increased 2.5 to 3.7-fold by three or more preceding or concomitant stressful events (Table 3). Adjusting for age, sex, education, body mass index, smoking status, alcohol intake and physical activity did not modify the effect of stressful events on allergies.

**Table 3:** Associations of allergies with stressful life events in 4002 Chinese people

Exposure	Odds of allergies (odds ratio, 95% confidence interval)^a^	
	Crude	Adjusted^**b**^
**Categorical variable**		
0 total stressful events	1.00	1.00
1 or 2 total stressful events	1.66 (1.43–1.94)***	1.65 (1.41–1.93)***
3 or more total stressful events	3.07 (2.54–3.73)***	3.10 (2.55–3.79)***
**Continuous variable**		
Total number of stressful events	1.27 (1.21–1.33)***	1.27 (1.21–1.33)***
Number of disease or death events	1.96 (1.70–2.26)***	1.95 (1.69–2.25)***
Number of conflict events	1.41 (1.28–1.56)***	1.39 (1.26–1.54)***
Number of life changing or other events	1.27 (1.19–1.36)***	1.27 (1.19–1.37)***

***P < 0.001, indicate significant odds ratios compared with reference. ^a^For models with continuous variable, odds ratios are the odds of allergies with increase in one stressful event. ^b^Adjusted for age, sex, education, body mass index, smoking status, alcohol intake and physical activity

### Stress management skills

Table 4 presents the independent associations between total stress management skills and 8 specific aspects and allergy risk in the study population. For all participants, data showed that the total stress management skills were significant negatively associated with allergies (OR, 0.74; 95% CI, 0.57–0.95), even after adjusting for age, sex, education, body mass index, smoking status, alcohol intake and physical activity. Significant decreased allergic risks were also observed among individuals who answered sometimes or often for “concentrate on pleasant thoughts at bedtime”, “pace myself to prevent tiredness”, “get enough sleep”, “take some time for relaxation each day” and “Balance time between work and play” (adjusted ORs ranged from 0.54 to 0.81, Table 4). No significant association with allergic risk was found for “accept those things in my life that I cannot change”, “use specific methods to control my stress” and “practise relaxation or meditation for 15-20 min daily”, despite a tendency towards a reduced allergic risk (ORs of good or moderate management skills are all negative compared with poor skills).

**Table 4:** Adjusted odds ratios and 95% confidence intervals for stress management skills in all participants and in subjects stratified by stressful life events

	All participants			Participants with stressful life events			Participants without stressful life events		
Variable	Case participants (1340)	Control participants (2662)	Adjusted odds^**a**^	Case participants (1000)	Control participants (1595)	Adjusted odds^**a**^	Case participants (340)	Control participants (1067)	Adjusted odds^**a**^
	***N*** (%)	***N*** (%)	(odds ratio, 95% confidence interval)	***N*** (%)	***N*** (%)	(odds ratio, 95% confidence interval)	***N*** (%)	N (%)	(odds ratio, 95% confidence interval)
**Total stress management skills**									
Poor	217 (16.2)	351 (13.2)	1	181 (18.1)	239 (15.0)	1	36 (10.6)	112 (10.5)	1
Moderate	926 (69.1)	1796 (67.5)	0.92 (0.76–1.12)	690 (69.0)	1079 (67.6)	0.92 (0.74–1.16)	236 (69.4)	717 (67.2)	1.17 (0.76–1.78)
Good	197 (14.7)	515 (19.3)	0.74 (0.57–0.95)*	129 (12.9)	277 (17.4)	0.71 (0.53–0.97)*	68 (20.0)	238 (22.3)	1.18 (0.72–1.96)
**Stress management skill items**									
**Accept those things in my life that I cannot change**									
Rarely or never	72 (5.4)	123 (4.6)	1	58 (5.8)	72 (4.5)	1	14 (4.1)	51 (4.8)	1
Sometimes	350 (26.1)	814 (30.6)	0.74 (0.54–1.03)	279 (27.9)	496 (31.1)	0.70 (0.47–1.02)	71 (20.9)	318 (29.8)	0.90 (0.46–1.74)
Often or always	918 (68.5)	1725 (64.8)	0.95 (0.69–1.30)	663 (66.3)	1027 (64.4)	0.82 (0.56–1.19)	255 (75.0)	698 (65.4)	1.61 (0.85–3.04)
**Use specific methods to control stress**									
Rarely or never	56 (4.2)	91 (3.4)	1	45 (4.5)	55 (3.4)	1	11 (3.2)	36 (3.4)	1
Sometimes	452 (33.7)	861 (32.3)	0.93 (0.65–1.33)	346 (34.6)	550 (34.5)	0.82 (0.54–1.26)	106 (31.2)	311 (29.1)	1.24 (0.60–2.60)
Often or always	832 (62.1)	1710 (64.2)	0.92 (0.64–1.31)	609 (60.9)	990 (62.1)	0.84 (0.56–1.28)	223 (65.6)	720 (67.5)	1.26 (0.61–2.60)
**Concentrate on pleasant thoughts at bedtime**									
Rarely or never	179 (13.4)	239 (9.0)	1	140 (14.0)	159 (10.0)	1	39 (11.5)	80 (7.5)	1
Sometimes	741 (55.3)	1461 (54.9)	0.73 (0.58–0.90)**	569 (56.9)	883 (55.4)	0.78 (0.60–1.01)	172 (50.6)	578 (54.2)	0.66 (0.43–1.02)
Often or always	420 (31.3)	962 (36.1)	0.67 (0.53–0.85)**	291 (29.1)	553 (34.7)	0.67 (0.51–0.89)**	129 (37.9)	409 (38.3)	0.79 (0.50–1.25)
**Pace myself to prevent tiredness**									
Rarely or never	342 (25.5)	521 (19.6)	1	275 (27.5)	320 (20.1)	1	67 (19.7)	201 (18.8)	1
Sometimes	506 (37.8)	1011 (38.0)	0.80 (0.67–0.96)*	380 (38.0)	611 (38.3)	0.75 (0.61–0.93)**	126 (37.1)	400 (37.5)	0.97 (0.68–1.37)
Often or always	492 (36.7)	1130 (42.4)	0.76 (0.63–0.91)**	345 (34.5)	664 (41.6)	0.67 (0.54–0.83)***	147 (43.2)	466 (43.7)	1.14 (0.80–1.62)
**Get enough sleep**									
Rarely or never	68 (5.1)	80 (3.0)	1	58 (5.8)	49 (3.1)	1	10 (2.9)	31 (2.9)	1
Sometimes	555 (41.4)	858 (32.2)	0.81 (0.57–1.14)	439 (43.9)	608 (38.1)	0.63 (0.42–0.94)*	116 (34.1)	250 (23.4)	1.55 (0.72–3.33)
Often or always	717 (53.5)	1724 (64.8)	0.54 (0.39–0.77)***	503 (50.3)	938 (58.8)	0.48 (0.32–0.72)***	214 (62.9)	786 (73.7)	0.97 (0.46–2.05)
**Take some time for relaxation each day**									
Rarely or never	87 (6.5)	95 (3.6)	1	71 (7.1)	58 (3.6)	1	16 (4.7)	37 (3.5)	1
Sometimes	558 (41.6)	1064 (40.0)	0.63 (0.46–0.86)**	422 (42.2)	684 (42.9)	0.53 (0.37–0.78)**	136 (40.0)	380 (35.6)	0.95 (0.50–1.79)
Often or always	695 (51.9)	1503 (56.5)	0.59 (0.43–0.82)**	507 (50.7)	853 (53.5)	0.55 (0.37–0.80)**	188 (55.3)	650 (60.9)	0.87 (0.46–1.64)
**Balance time between work and play**									
Rarely or never	84 (6.3)	122 (4.6)	1	72 (7.2)	86 (5.4)	1	12 (3.5)	36 (3.4)	1
Sometimes	632 (47.2)	1034 (38.8)	0.95 (0.71–1.29)	491 (49.1)	662 (41.5)	0.93 (0.66–1.31)	141 (41.5)	372 (34.9)	1.24 (0.61–2.50)
Often or always	624 (46.6)	1506 (56.6)	0.69 (0.51–0.94)*	437 (43.7)	847 (53.1)	0.68 (0.48–0.96)*	187 (55.0)	659 (61.8)	1.03 (0.51–2.08)
**Practise relaxation or meditation for 15–20 min daily**									
Rarely or never	146 (10.9)	223 (8.4)	1	116 (11.6)	142(8.9)	1	30 (8.8)	81 (7.6)	1
Sometimes	578 (43.1)	1099 (41.3)	0.87 (0.68–1.10)	433 (43.3)	667 (41.8)	0.84 (0.64–1.11)	145 (42.6)	432 (40.5)	0.99 (0.62–1.60)
Often or always	616 (46.0)	1340 (50.3)	0.81 (0.64–1.03)	451 (45.1)	786 (49.3)	0.79 (0.59–1.05)	165 (48.5)	554 (51.9)	0.99 (0.61–1.59)

*P < 0.05, **P < 0.01, ***P < 0.001, indicate significant odds ratios compared with reference. ^a^Adjusted for age, sex, education, body mass index, smoking status, alcohol intake and physical activity

Results from the stratified analyses are presented in Table 4. Similar to the results for all participants, using one or more stress management skills was significantly or borderline significantly associated with decreased allergic risk among people with stressful events, especially “pace myself to prevent tiredness”,“get enough sleep”,“take some time for relaxation each day”, even sometimes performing them will achieve a good result. Notably, there was no significant association between stress management skills and allergy risk among people without stressful events.

### Combined effect of stressful life events and stress management skills

We further analyzed the combined effects of stressful events and stress management skills on the risk of allergic diseases (Table 5). There was a significant linear trend for the risk of allergies from good stress management skills with no stressful events to poor stress management skills with stressful events (*P* < 0.001, Table S2). Although an interaction in the multiplicative model was not observed, significant additive interactions were found between stressful events and total stress management skills (*P* = 0.255 and 0.006, respectively). The results for “concentrate on pleasant thoughts at bedtime” and “balance time between work and play” were similar to those for total stress management skills (Table 5). Interaction was significant in both multiplicative and additive models for “pace myself to prevent tiredness” (*P* = 0.023 and < 0.001, respectively).

**Table 5:** Interaction between stressful life events and stress management skills. in southern Chinese allergy study

Stress management skills	*P* for interaction	
	Multiplicative	Additive
Total stress management skills	0.255	**0.006**
Accept those things in my life that I cannot change	**0.019**	0.113
Use specific methods to control stress	0.936	0.685
Concentrate on pleasant thoughts at bedtime	0.286	**0.006**
Pace myself to prevent tiredness	**0.023**	**<0.001**
Get enough sleep	0.802	0.193
Take some time for relaxation each day	0.812	0.517
Balance time between work and play	0.489	**0.008**
Practise relaxation or meditation for 15–20 min daily	0.699	0.170

Significant differences are highlighted in bold

## Discussion

There is a great body evidence of stress/stress events on onset or aggravation allergic diseases [9, 26], so management interventions are focused on individual, family, school, medical provider, et al. [46, 47]. However, few studies explore the relationship between stress management skills and allergic diseases. Here we performed a survey on possible risk factors for the development of allergic diseases in a large sample population of 28,144, and then conduct a case-control study to explore the allergy risk with stressful life events, stress management skills and their combination effects.

First of all, in order not to be affected by any illness or disease, we analyzed basic characteristics of cases and controls in two groups (with or without self-reported illness or injury). The results show no significant difference of age, Sex, education, BMI, smoking status and alcohol intake between case and control participants except for physical activity which allergic participants were unlikely to choose. The reason may due to the effect of physical activity on the induction and exacerbation of the allergic symptoms [48]. Another Korea research also proved that regular vigorous PA (physical activity) may aggravate AR (allergic rhinitis) symptoms by subjective or objective factors [49]. But there are different opinions and results. For example, Counil FP’s study reported that children with mild-to-moderate asthma can tolerate an exercise training program well which can improve their both aerobic and anaerobic fitness [50]. Another randomized controlled study shows that winter exercise reduces allergic airway inflammation [51]. In fact, positive regular physical exercise can not only prevent cardiovascular disease, diabetes, cancer, hypertension, obesity, depression and osteoporosis and other several chronic diseases, but also prevent allergic diseases [48], which requires a suitable intensity and style.

Then, in the present study we found 65.8% of 28,144 participants reported one or more stressful events during their lifetime, which is similar to previous reports [27]. People with allergies were likely to experience more stressful life events compared with controls (the risk increased 2.5 to 3.7-fold by experiencing three or more preceding or concomitant stressful events). This positive association is consistent with outcomes from other investigations. A prospective study showed that the illness of a family member, marital problems, divorce or separation, and conflicts with a supervisor were associated with the onset of asthma [29]. A Norwegian cross-sectional study found that exposure to violence and stressful events excite or aggravate asthma among 15-year-old adolescents [52]. It is known that diseases, bereavements, conflicts in personal or parental relationships are common stress events [26–29]. In our study, life event selection was based on well-known life event scales [37]. The results showed that lifestyle changes, overloading the individual, economic plight, living environment change, a criminal or civil penalty were also associated with allergic episodes. Another study confirmed this by showing that children who spent a year living abroad were more sensitive than children in control group, suggesting stress related to living environment change may promote the development of atopy [53]. However, a cross-sectional design did not allow assessment of the causality or directionality of relationships. Diagnosed allergies might also lead to increased stress and stress events. R Lietzén,et al. [29] has conducted a prospective cohort study among 16,881 participants free of diagnosed asthma. During the 2 years’ follow-up period, 192 incident asthma cases were identified, and high total exposure to stressful life events predicted asthma attacks (hazard ratio 1.96, 95% CI 1.22–3.13). More prospective studies are warranted on stressful events and allergies.

Several popular approaches dealing with stress were used [54], such as raising awareness of stressors and coping styles, relaxation techniques, anger management and anxiety reduction techniques, incorporating healthy diet and regular physical exercise, managing time, et al. However, up to present, it’s still difficult to determine individual stress risk or the effectiveness of individual stress management/reduction strategies. There are a few profile that can access these factors comprehensively. We chose the HPLP-II, which assesses daily stress management lifestyle behaviors and establishes the individuals’ ability to acknowledge sources of stress and control mechanisms. Our results indicates that people with allergic diseases have worse stress management skills, especially in the areas of “concentrate on pleasant thoughts at bedtime”, “pace myself to prevent tiredness”, “get enough sleep”, “take some time for relaxation each day” and “balance time between work and play”, which are skills that relax and reduce fatigue. As we know, relaxation techniques have been shown to significantly reduce activity of stress-responsive systems and thus setting the stage for either active stress management or regeneration [34, 55]. Further stratified analyses show that stress management skills are significantly associated with decreased allergy risk only among participants who experienced stressful events, but it’s worth noting that similar results are not observed among people without stressful events. This suggests that stress management skills are likely to be needed when stressful events are being experienced, but could be omitted when there are no stressful events, and this has not been reported hitherto. Our finding gives suggestive guidance for the timing of stress management skills training among allergic patients, which means when allergic patients have never experienced a trouble from a stressful event experience, stress management skills won’t have effect on the improvement of the condition.

The results are further verified when we explore the combined effect of stressful events and stress management skills on allergic diseases (Table S2). Allergy risk increased 2.06-fold (90% CI 1.53–3.04) when people experience stressful events with poor stress management skills compared to participants experience no stressful events and meanwhile with good stress management skills. And a linear trend for the risk of allergies from good stress management skills with stressful events to poor stress management skills with stressful events can be observed. However, in participants with no stressful events, there is no significant risk change (*P* > 0.05) between participants with good or poor stress management skills. The further study has proven the additive interactions between stressful events and stress management skills (Table 5). From what has been discussed, we can get instructive conclusions that among people with stressful life events, they can take some stress management skills to prevent or stable the condition of allergic disease, such as “concentrate on pleasant thoughts at bedtime”, “pace myself to prevent tiredness”, “balance time between work and play” and “balance time between work and play”. This has realistic guiding significance to allergic patients.

Relationships of the central nervous stress system and peripheral immune system have been studied in the past 50 years. Psychoneuroimmunology (PNI) focuses on neuroendocrine-immune system interactions in multidimensional psychobehavioral. The related mechanisms are mainly concluded in three aspects: the hypothalamic-pituitary adrenocortical (HPA) system, which coordinates the release of corticotropin (ACTH), endorphins, and glucocorticoids; the sympathetic nervous system (SNS) with two branches of the adrenal medullary system (adrenaline) and sympathetic nerve fibers (noradrenaline), can reach almost every part of the body; and the parasympathetic nervous system (PSNS) with acetylcholine as the main neurotransmitter, has anti-inflammatory and immunosuppressive effects [56]. The exact biological mechanisms are not understood, the potential mechanisms may be as follows: Perception of stress activates the HPA system, which promotes secretion of corticotrophin-releasing hormone (CRH) and then induces secretion of adrenocorticotrophic hormone (ACTH) by the anterior lobe of the pituitary lobe, ACTH activates the secretion of catecholamines (adrenalin and noradrenalin), then suppresses IL-12 production [57], or affects Th2 cells thus increasing the production of interleukin cytokines [58]. Evidences show that central nervous system (CNS) can also affect the organ function through noradrenalin and acetylcholine, these neurotransmitters can directly innervate the important organs and systems related to the immune system such as the liver, spleen, skin, et al. [59]. Reports shows that adolescents with asthma who experienced more stress have higher levels of interleukin 5 (IL-5) and interferon-c (IFN-c), which associated with type 2 and type 1 immune responses, respectively [60]. In animals with chronic stress, elevated glucocorticoid levels and increased cholinergic system function can also be observed [61]. These alterations of adrenal function and subsequent cytokines profiles may thus link chronic stress to immunity changes favouring atopy. It is worth noting that cognitive behavioral therapy, such as mind-body therapies (including Taihi, Qigong, yoga, mindfulness-based stress reduction) and wellness education can beneficially influence the readout parameters of the immune system [62].

New fields of investigation links stress with allergic diseases are flourishing. For example, among genes that regulate stress response, the role of allelic variation has been reported, as well as stress-induced DNA methylation patterns and gene expression changes [13]. A recent study investigating whether leukocyte telomerase activity changes in response to acute psychological stress showed that increased telomerase activity was not associated with changes in the number or percentage of monocytes, lymphocytes, and specific T cell types, but was associated with greater cortisol increases [63]. Intestinal permeability and intestinal flora are also one of the research hotspots [64], and probiotics have been reported to modulate immune responses and their supplementation as a preventive intervention affects the development of sensitization and allergies [65]. So further studies are needed to demonstrate a direct link between clinical improvement and immunological changes after psychological interventions in allergic diseases.

### Limitations

First, our study was conducted in 2012–2013, the data may not be up-to-date that cannot truly represent the current situation. Relevant research may also be advanced. But there is no denying that our data from such a large sample in southern China is very precious and can represent the holistic picture. With the improvement of living standard, the prevalence of allergies and asthma have been increased during the past 9 years. The World Health Organization (WHO) estimates that 400 million people in the world suffer from allergic rhinitis, and 300 million from asthma [51], psychosocial stress (means the self-reported sensation of tension, irritability, nervousness, anxiety or sleeplessness [8] associated with poor health, family relationships, living arrangements, finance, work and stressful life events) [9–12] are becoming more and more important in onset or exacerbations of allergic diseases. We have reviewed a large body of literature include those have been published recently, and find there are few studies explore the stress management effects on allergic disease, when we enter the following search strategy as ((((allergic [Title/Abstract]) OR (asthma [Title/Abstract])) OR (atopic [Title/Abstract]))) AND (stress management [Title/Abstract]), there are only 14 articles retrieved, and four of which have been published after 2013. Most of them are reviews, or mention “stress management” in discussion. One RCT research public in 2013 used a “Cognitive behavioural stress management programmes (CBS)”, not stress management skills can be used in daily life. So, the state of the science still cannot identify the effectiveness of individual stress management strategies. Our case-control study based on 28,144 samples sought to examine the link between stress management skills and allergies, as well as it’s interaction effects with stressful events on allergy risk, which haven’t been reported before. Besides, our finding gives suggestive guidance for the timing of stress management skills training among allergic patients, which was innovative and would be the first report.

Second, we relied on questionnaire in the study, which is prone to several sources of bias [66, 67] although our study has included large population-based sample and high participation rates. As we know, self-reports of questionnaire is a pragmatic and efficient option of data collection for large epidemiological studies. And self-reported data is non-expensive and do not involve complicated field logistics or invasive procedures. This is the common method used in the epidemiological research. And we had taken measures to avoid the bias for accuracy data.

For selection bias: epidemiological studies can be affected by selection bias. In this study, we have included six cities randomly selected in Guangdong province, and then selected one or two convenient areas (e.g. schools, companies, government agencies or factories) in each city for cluster sampling. The sample size is reasonably large, and the response rate is relatively high (83.01%). Thus, a selection bias is unlikely. Furthermore, we have matched the age and sex that may reduce the bias in reporting the disease [68], and also bias caused by sex differences in the prevalence of the disease.

For recall bias: The recall biases in our manuscript are mainly on the allergic disease and the stressful life events. However, enterprises, factories or government unit would hold routine health examinations for employees every year. We evaluated these medical histories getting from each unit managers (at least one-year medical examination report between 2012 and 2013 for each participant) to minimize the diseases recall bias. Furthermore, we select life events based on well-known life event scales [36]. Study participants indicated (yes/no) their experience of 14 major events, which are connected with disease or death of family members or close friends; conflicts in personal relationships or life changes. These events have a strong impact on emotions, so would be remembered reliably by participants. They are likely to be recalled more accurately than other events [69]. And exclusion of cases who completed questionnaires after five-year post diagnosis is done to minimize recall bias and reverse. Thus, recall bias could have influenced our estimates only to a minimal extent.

## Conclusions

Our case-control study based on 28,144 samples confirmed a strong positive association between stressful life events and allergies, and a significantly inverse relationship between stress management skills and allergies. We have also observed antagonism effects of stress management skills with stressful events on allergy risk. Stress management skills only effect in allergic patients who had experienced stressful life events. This finding gives suggestive guidance for the timing of stress management skills training among allergic patients, which was innovative and would be the first report. Further studies are warranted to characterize the exact preventative role of stress management skills in the etiology or progression of allergies.

## Supplementary Information

**Additional file 1: Table S1.** Stress management scale items and scoring methods.

**Additional file 2: Table S2.** Combined effects of stressful life events and stress management skills in southern urban Chinese allergy study.

**Additional file 3: Questionnaire 1.** Life Events and Allergic Disease Survey Scale.

**Additional file 4: Questionnaire 2.** Lifestyle Profile II.

## Data Availability

All data generated or analyzed during this study are included in this published article.

## Abbreviations

OR: odds ratio
CI: Confidence interval
DM: diabetic mellitus
HPLP-II: Health Promoting Lifestyle Profile II
MOCS: One scale measurement of Current Status
IgE: immunoglobulin-E
PNI: psychoneuroimmunology
HPA: hypothalamic-pituitary- adrenocortical
SNS: sympathetic nervous system
PSNS: parasympathetic nervous system
CRH: corticotrophin-releasing hormone
ACTH: adrenocorticotrophic hormone
CNS: central nervous system
