# Supplementary material for: Association of stress management skills and stressful life events with allergy risk: a case-control study in southern China
Source: BMC Public Health. 2021 Jun 30;21:1279. doi: 10.1186/s12889-021-11333-3 (PMC8247235; doi:10.1186/s12889-021-11333-3)
Supplement: Supplementary file 1 — Additional file 1: Table S1. Stress management scale items and scoring methods. [file 12889_2021_11333_MOESM1_ESM.docx]

| **Table S1 Stress management scale items and scoring methods** | | | | |
| --- | --- | --- | --- | --- |
| **Variable** | rarely/never | sometimes | often | always |
| Accept those things in my life  which I cannot change | 1 | 2 | 3 | 4 |
| Use specific methods to  control my stress | 1 | 2 | 3 | 4 |
| Concentrate on pleasant  thoughts at bedtime | 1 | 2 | 3 | 4 |
| Pace myself to prevent tiredness | 1 | 2 | 3 | 4 |
| Get enough sleep | 1 | 2 | 3 | 4 |
| Take some time for relaxation each day | 1 | 2 | 3 | 4 |
| Balance time between work  and play | 1 | 2 | 3 | 4 |
| Practice relaxation or meditation for 15-20 min daily | 1 | 2 | 3 | 4 |
| Items score using a four-point Likert scale as 1 (never or rarely), 2 (sometimes), 3 (often), 4 (always);Total stress management skill score ranges from 8 to 32, higher scores represent more engagement in stress management behaviour. | | | | |
