# Supplementary material for: Association of stress management skills and stressful life events with allergy risk: a case-control study in southern China
Source: BMC Public Health. 2021 Jun 30;21:1279. doi: 10.1186/s12889-021-11333-3 (PMC8247235; doi:10.1186/s12889-021-11333-3)
Supplement: Supplementary file 2 — Additional file 2: Table S2. Combined effects of stressful life events and stress management skills in southern urban Chinese allergy study. [file 12889_2021_11333_MOESM2_ESM.docx]

| **Table S2 Combined effects of stressful life events and stress management skills in southern urban Chinese allergy study** | | | | |
| --- | --- | --- | --- | --- |
| **Stress management skills** | **Stressful  life events** | **Cases** | **Controls** | **Odds of allergies (OR,95% CI)** |
|  |  | **N(%)** | **N(%)** | **Adjusted#** |
| **Total stress management skills** |  |  |  |  |
| Good  Moderate  Poor  Good  Moderate  Poor | No No No Yes Yes Yes | 68(5.1) 236(17.6) 36(2.7) 129(9.6) 690(51.5) 181(13.5) | 238(8.9) 720(27.0) 109(4.1) 277(10.4) 1079(40.5) 239(9.0) | 1 1.05(0.77-1.44) 0.95(0.59-1.53) 1.62(1.15-2.29)** 2.06(1.54-2.75)*** 2.16(1.53-3.04)*** |
| P value for trend¶ |  |  |  | **0.000** |
| **Accept those things in my life  which I cannot change** |  |  |  |  |
| Often/always  Sometimes  Rarely/never  Often/always  Sometimes  Rarely/never | No No No Yes Yes Yes | 255(19.0) 71(5.3) 14(1.0) 663(49.5) 279(20.8) 58(4.3) | 698(26.2) 318(11.9) 51(1.9) 1027(38.6) 496(18.6) 72(2.7) | 1 0.58(0.43-0.79)*** 0.70(0.38-1.29) 1.75(1.47-2.09)*** 1.46(1.19-1.80)*** 2.07(1.41-3.05)*** |
| P value for trend¶ |  |  |  | **0.000** |
| **Use specific methods to  control my stress** |  |  |  |  |
| Often/always  Sometimes  Rarely/never  Often/always  Sometimes  Rarely/never | No No No Yes Yes Yes | 223(16.6) 106(7.9) 11(0.8) 609(45.4) 346(25.8) 45(3.4) | 720(27.0) 311(11.7) 36(1.4) 990(37.2) 550(20.7) 55(2.1) | 1 1.04(0.79-1.36) 0.83(0.41-1.68) 1.97(1.64-2.37)*** 1.89(1.54-2.32)*** 2.28(1.48-3.51)*** |
| P value for trend¶ |  |  |  | **0.000** |
| **Concentrate on pleasant  thoughts at bedtime** |  |  |  |  |
| Often/always  Sometimes  Rarely/never  Often/always  Sometimes  Rarely/never | No No No Yes Yes Yes | 129(9.6) 172(12.8) 39(2.9) 291(21.7) 569(42.5) 140(10.4) | 409(15.4) 578(21.7) 80(3.0) 553(20.8) 883(33.2) 159(6.0) | 1 0.87(0.67-1.13) 1.32(0.85-2.05) 1.64(1.28-2.10)*** 1.88(1.50-2.36)*** 2.40(1.76-3.27)*** |
| P value for trend¶ |  |  |  | **0.000** |
| **Pace myself to prevent tiredness** |  |  |  |  |
| Often/always  Sometimes  Rarely/never  Often/always  Sometimes  Rarely/never | No No No Yes Yes Yes | 147(11.0) 126(9.4) 67(5.0) 345(25.7) 380(28.4) 275(20.5) | 466(17.5) 400(15.0) 201(7.6) 664(24.9) 611(23.0) 320(12.0) | 1 0.92(0.70-1.21) 0.93(0.67-1.31) 1.65(1.31-2.07)*** 1.80(1.43-2.26)*** 2.38(1.85-3.06)*** |
| P value for trend¶ |  |  |  | **0.000** |
| **Get enough sleep** |  |  |  |  |
| Often/always  Sometimes  Rarely/never  Often/always  Sometimes  Rarely/never | No No No Yes Yes Yes | 214(16.0) 116(8.7) 10(0.7) 503(37.5) 439(32.8) 58(4.3) | 786(29.5) 250(9.4) 31(1.2) 938(35.2) 608(22.8) 49(1.8) | 1 1.65(1.26-2.16)*** 1.05(0.50-2.19) 1.98(1.64-2.39)*** 2.54(2.08-3.10)*** 3.97(2.62-6.02)*** |
| P value for trend¶ |  |  |  | **0.000** |
| **Take some time for relaxation each day** |  |  |  |  |
| Often/always  Sometimes  Rarely/never  Often/always  Sometimes  Rarely/never | No No No Yes Yes Yes | 188(14.0) 136(10.1) 16(1.2) 507(37.8) 422(31.5) 71(5.3) | 650(24.4) 380(14.3) 37(1.4) 853(32.0) 684(25.7) 58(2.2) | 1 1.15(0.89-1.49) 1.25(0.67-2.32) 2.05(1.68-2.50)*** 1.97(1.60-2.42)*** 3.59(2.42-5.32)*** |
| P value for trend¶ |  |  |  | **0.000** |
| **Balance time between work  and play** |  |  |  |  |
| Often/always  Sometimes  Rarely/never  Often/always  Sometimes  Rarely/never | No No No Yes Yes Yes | 187(14.0) 141(10.5) 12(0.9) 437(32.6) 491(36.6) 72(5.4) | 659(24.8) 372(14.0) 36(1.4) 847(31.8) 662(24.9) 86(3.2) | 1 1.26(0.97-1.63) 1.04(0.53-2.05) 1.82(1.49-2.23)*** 2.44(1.99-2.99)*** 2.58(1.79-3.72)*** |
| P value for trend¶ |  |  |  | **0.000** |
| **Practice relaxation or meditation for 15-20 min daily** |  |  |  |  |
| Often/always  Sometimes  Rarely/never  Often/always  Sometimes  Rarely/never | No No No Yes Yes Yes | 165(12.3) 145(10.8) 30(2.2) 451(33.7) 433(32.3) 116(8.7) | 554(20.8) 432(16.2) 81(3.0) 786(29.5) 667(25.1） 142(5.3) | 1 1.05(0.81-1.37) 1.06(0.67-1.69) 1.91(1.55-2.37)*** 2.01(1.62-2.49)*** 2.38(1.75-3.24)*** |
| P value for trend¶ |  |  |  | **0.000** |
| #Adjusted for age, sex, education, body mass index, smoking status, alcohol intake and physical activity. ¶Significant differences of P value for trend were highlighted in bold. | | | | |
