# Supplementary material for: Association of stress management skills and stressful life events with allergy risk: a case-control study in southern China
Source: BMC Public Health. 2021 Jun 30;21:1279. doi: 10.1186/s12889-021-11333-3 (PMC8247235; doi:10.1186/s12889-021-11333-3)
Supplement: Supplementary file 3 — Additional file 3: Questionnaire 1. Life Events and Allergic Disease Survey Scale. [file 12889_2021_11333_MOESM3_ESM.docx]

**Life Events and Allergic Disease Survey Scale**

1. Name: ; Sex: ; Nation: ; Ancestral home: .
2. Date of birth: ; Have lived in Guangdong province for years.

Hight: cm; Weight: Kg.

1. Your highest educational attainment:

□Primary school and below; □ Junior high school; □Senior high school;

□Junior college; □Undergraduate college; □Graduate school.

1. Marital status:

□Spinsterhood; □Married; □Divorced ; □Other status.

1. Profession:

□Professional and technical personnel; □Personnel of State organs, Party and mass organizations, enterprises and public institutions; □Servicemen; □Students; □Production personnel in the fields of agriculture, forestry, animal husbandry and water conservancy;□Operators of production and transport equipment and other related personnel;□Business and service personnel;□Freelancer;□No occupation (housewife, etc.) ;□Other profession: .

1. Do you smoke? □No □Yes, it has been years since you started smoking.

□Quit smoking, quit smoking for years.

1. Do you drink alcohol? □Never;□Seldom;□Sometimes;□Often;□Always.
2. Have you experienced the following events? (Multiple options)

|  | Self-reported events | Yes | No |
| --- | --- | --- | --- |
| 1 | Personal injury or illness | 1 | 0 |
| 2 | Severe disease of family member or close friend | 1 | 0 |
| 3 | Death of family member or close friend | 1 | 0 |
| 4 | Interpersonal disharmony | 1 | 0 |
| 5 | Family discord | 1 | 0 |
| 6 | Marital disruption | 1 | 0 |
| 7 | Trouble from children | 1 | 0 |
| 8 | Split up from boyfriend or girlfriend | 1 | 0 |
| 9 | Overwork | 1 | 0 |
| 10 | Economic plight | 1 | 0 |
| 11 | Career change | 1 | 0 |
| 12 | Living environment change | 1 | 0 |
| 13 | Lifestyle change | 1 | 0 |
| 14 | Suffer a criminal or civil penalty | 1 | 0 |

1. Do you have any Allergic diseases? (Multiple options)

□Asthma: Have you ever had asthma?

□Allergic rhinitis: Have you ever had ‘hay fever’ or other allergic nasal symptoms (sneezing, nasal itching, blocked nose or runny nose) in the absence of a cold or flu, e.g. from pollen or animals?

□Atopic dermatitis: Have you ever had symptoms of itchy rash called atopic eczema or eczema localized to flexural regions (such as folds of the elbows, behind the knees), facial, or generalized to the body?

If so, has a doctor diagnosed the disease? .

1. Do you have any other chronic diseases? (Multiple options)

Cardiovascular and cerebrovascular diseases:

□Heart disease; □Hypertension ; □Cerebrovascular disease; □Arteriosclerosis;

Metabolic or endocrine diseases:

□Diabetes; □Hyperlipidemia; □Hyperuricemia; □Thyroid disorder;

Respiratory system diseases:

□Chronic bronchitis; □Emphysema; □Chronic laryngitis; □Tuberculosis;

Digestive system diseases:

□Fatty liver; □Cholecystitis;□Chronic hepatitis ; □Cirrhosis; □Hemorrhoids;

□Chronic gastritis; □Stomach, duodenal ulcer.

Urinary system diseases:

□Chronic nephritis; □Kidney failure; □Prostate disease.

Rheumatic or autoimmune diseases:

□Rheumatism; □Systemic lupus erythematosus; □Sicca syndrome;

Gynecological disease:

□Breast disease;□Gynecologic inflammation;

Mental and psychological diseases:

□Chronic insomnia; □Anxiety disorder ; □Depression.

Other diseases (please fill in) : .

If so, has a doctor diagnosed the disease? .

1. Is the disease currently cured? □ Cured; □ Not yet.

If not, is your condition well controlled?

□ Good control, no related symptoms; □ Poor control, recurrent symptoms

What medications are taking for the disease: .
