# Supplementary material for: Association of stress management skills and stressful life events with allergy risk: a case-control study in southern China
Source: BMC Public Health. 2021 Jun 30;21:1279. doi: 10.1186/s12889-021-11333-3 (PMC8247235; doi:10.1186/s12889-021-11333-3)
Supplement: Supplementary file 4 — Additional file 4: Questionnaire 2. Lifestyle Profile II. [file 12889_2021_11333_MOESM4_ESM.docx]

**Lifestyle Profile II**

DIRECTIONS: This questionnaire contains statements about your present way of life or personal habits.

Please respond to each item as accurately as possible, and try not to skip any item. Indicate the frequency with which you engage in each behavior by circling:

N for never, S for sometimes, O for often, or R for routinely

| 1 | Discuss my problems and concerns with people close to me. | N | S | O | R |
| --- | --- | --- | --- | --- | --- |
| 2 | Choose a diet low in fat, saturated fat, and cholesterol. | N | S | O | R |
| 3 | Report any unusual signs or symptoms to a physician or other health professional. | N | S | O | R |
| 4 | Follow a planned exercise program. | N | S | O | R |
| 5 | Get enough sleep. | N | S | O | R |
| 6 | Feel I am growing and changing in positive ways. | N | S | O | R |
| 7 | Praise other people easily for their achievements. | N | S | O | R |
| 8 | Limit use of sugars and food containing sugar (sweets). | N | S | O | R |
| 9 | Read or watch TV programs about improving health. | N | S | O | R |
| 10 | Exercise vigorously for 20 or more minutes at least three times a week (such as brisk walking, bicycling, aerobic dancing, using a stair climber). | N | S | O | R |
| 11 | Take some time for relaxation each day. | N | S | O | R |
| 12 | Believe that my life has purpose. | N | S | O | R |
| 13 | Maintain meaningful and fulfilling relationships with others. | N | S | O | R |
| 14 | Eat 6-11 servings of bread, cereal, rice and pasta each day. | N | S | O | R |
| 15 | Question health professionals in order to understand their instructions. | N | S | O | R |
| 16 | Take part in light to moderate physical activity (such as sustained walking 30-40 minutes 5 or more times a week). | N | S | O | R |
| 17 | Accept those things in my life which I can not change. | N | S | O | R |
| 18 | Look forward to the future. | N | S | O | R |
| 19 | Spend time with close friends. | N | S | O | R |
| 20 | Eat 2-4 servings of fruit each day. | N | S | O | R |
| 21 | Get a second opinion when I question my health care provider's advice. | N | S | O | R |
| 22 | Take part in leisure-time (recreational) physical activities (such as swimming, dancing, bicycling). | N | S | O | R |
| 23 | Concentrate on pleasant thoughts at bedtime. | N | S | O | R |
| 24 | Feel content and at peace with myself. | N | S | O | R |
| 25 | Find it easy to show concern, love and warmth to others. | N | S | O | R |
| 26 | Eat 3-5 servings of vegetables each day. | N | S | O | R |
| 27 | Discuss my health concerns with health professionals. | N | S | O | R |
| 28 | Do stretching exercises at least 3 times per week. | N | S | O | R |
| 29 | Use specific methods to control my stress. | N | S | O | R |
| 30 | Work toward long-term goals in my life. | N | S | O | R |
| 31 | Touch and am touched by people I care about. | N | S | O | R |
| 32 | Eat 2-3 servings of milk, yogurt or cheese each day. | N | S | O | R |
| 33 | Inspect my body at least monthly for physical changes/danger signs. | N | S | O | R |
| 34 | Get exercise during usual daily activities (such as walking during lunch, using stairs instead of elevators, parking car away from destination and walking). | N | S | O | R |
| 35 | Balance time between work and play. | N | S | O | R |
| 36 | Find each day interesting and challenging. | N | S | O | R |
| 37 | Find ways to meet my needs for intimacy. | N | S | O | R |
| 38 | Eat only 2-3 servings from the meat, poultry, fish, dried beans, eggs, and  nuts group each day. | N | S | O | R |
| 39 | Ask for information from health professionals about how to take good care of myself. | N | S | O | R |
| 40 | Check my pulse rate when exercising. | N | S | O | R |
| 41 | Practice relaxation or meditation for 15-20 minutes daily. | N | S | O | R |
| 42 | Am aware of what is important to me in life. | N | S | O | R |
| 43 | Get support from a network of caring people. | N | S | O | R |
| 44 | Read labels to identify nutrients, fats, and sodium content in packaged food. | N | S | O | R |
| 45 | Attend educational programs on personal health care. | N | S | O | R |
| 46 | Reach my target heart rate when exercising. | N | S | O | R |
| 47 | Pace myself to prevent tiredness. | N | S | O | R |
| 48 | Feel connected with some force greater than myself. | N | S | O | R |
| 49 | Settle conflicts with others through discussion and compromise. | N | S | O | R |
| 50 | Eat breakfast. | N | S | O | R |
| 51 | Seek guidance or counseling when necessary. | N | S | O | R |
| 52 | Expose myself to new experiences and challenges. | N | S | O | R |
